# Lessons from single-cell RNA sequencing of human islets

**DOI:** 10.1007/s00125-022-05699-1

**Published:** 2022-04-28

**Authors:** Mtakai Ngara, Nils Wierup

**Affiliations:** Lund University Diabetes Centre, Malmö, Sweden

**Keywords:** Alpha cell, Beta cell, Differential expression analysis, Ghrelin cell, Islet, Single-cell RNA sequencing, Type 2 diabetes mechanisms

## Abstract

Islet dysfunction is central in type 2 diabetes and full-blown type 2 diabetes develops first when the beta cells lose their ability to secrete adequate amounts of insulin in response to raised plasma glucose. Several mechanisms behind beta cell dysfunction have been put forward but many important questions still remain. Furthermore, our understanding of the contribution of each islet cell type in type 2 diabetes pathophysiology has been limited by technical boundaries. Closing this knowledge gap will lead to a leap forward in our understanding of the islet as an organ and potentially lead to improved treatments. The development of single-cell RNA sequencing (scRNAseq) has led to a breakthrough for characterising the transcriptome of each islet cell type and several important observations on the regulation of cell-type-specific gene expression have been made. When it comes to identifying type 2 diabetes disease mechanisms, the outcome is still limited. Several studies have identified differentially expressed genes, although there is very limited consensus between the studies. As with all new techniques, scRNAseq has limitations; in addition to being extremely expensive, genes expressed at low levels may not be detected, noise may not be appropriately filtered and selection biases for certain cell types are at hand. Furthermore, recent advances suggest that commonly used computational tools may be suboptimal for analysis of scRNAseq data in small-scale studies. Fortunately, development of new computational tools holds promise for harnessing the full potential of scRNAseq data. Here we summarise how scRNAseq has contributed to increasing the understanding of various aspects of islet biology as well as type 2 diabetes disease mechanisms. We also focus on challenges that remain and propose steps to promote the utilisation of the full potential of scRNAseq in this area.

**Graphical abstract:**

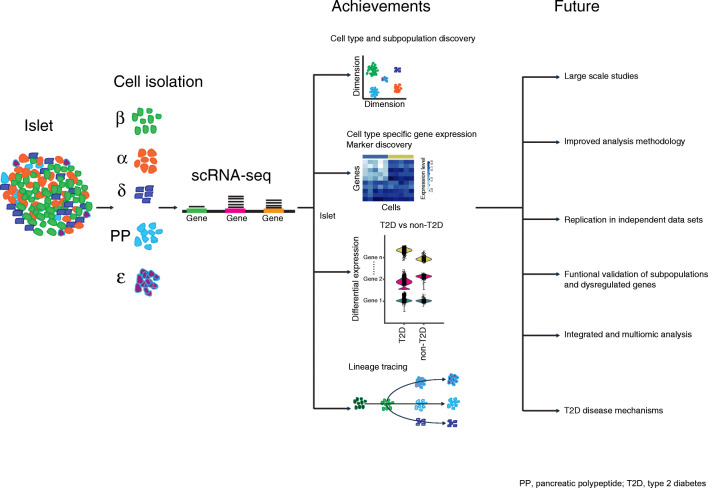

**Supplementary Information:**

The online version contains peer-reviewed but unedited supplementary material including a slide of the figure for download, which is available to authorised users at 10.1007/s00125-022-05699-1.

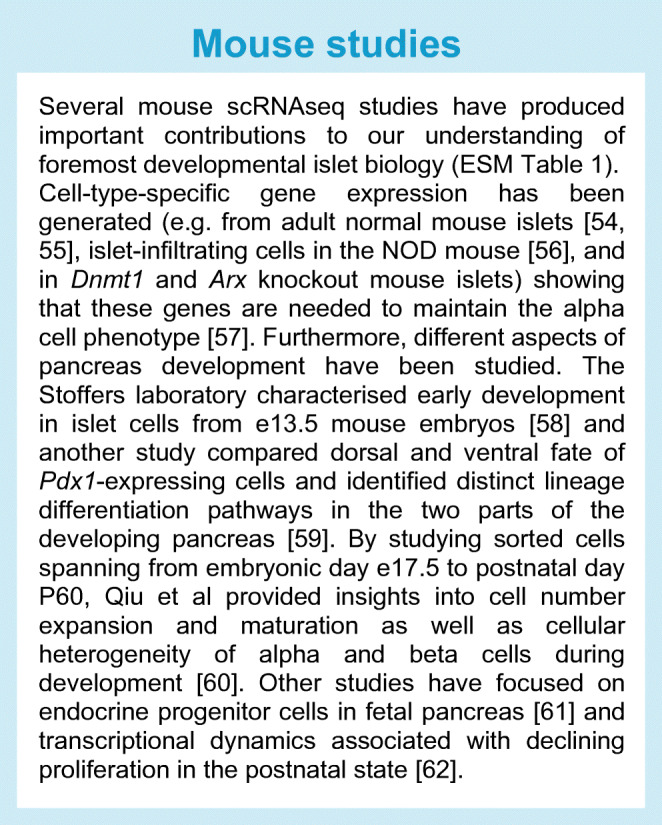

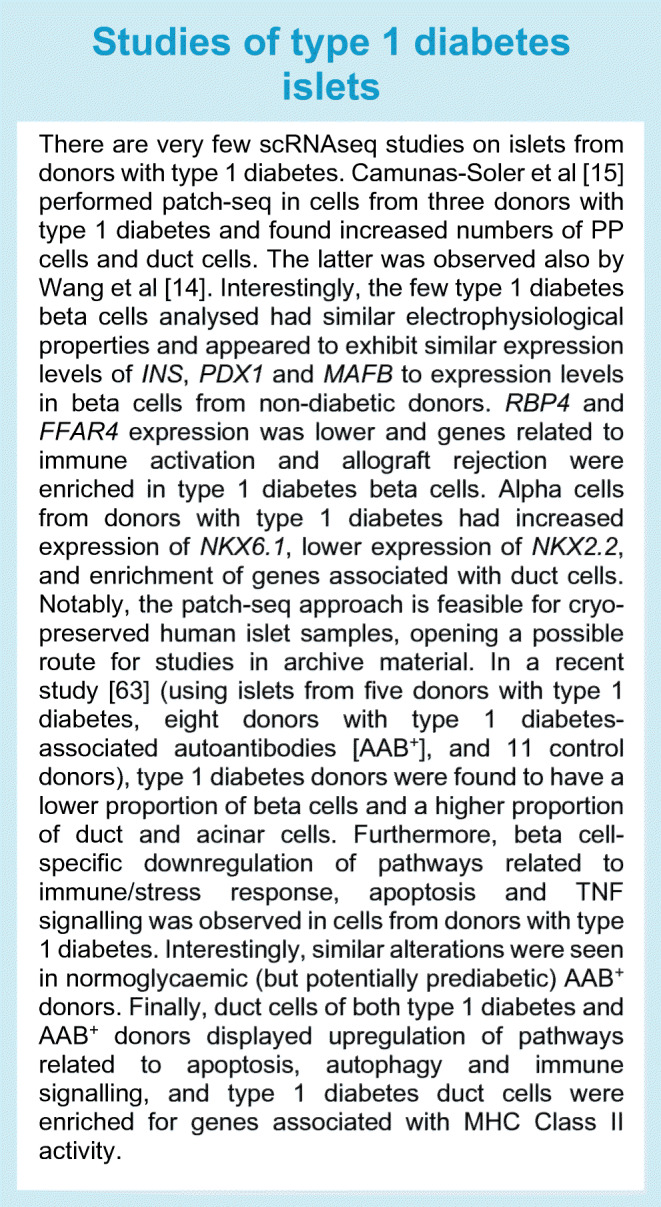

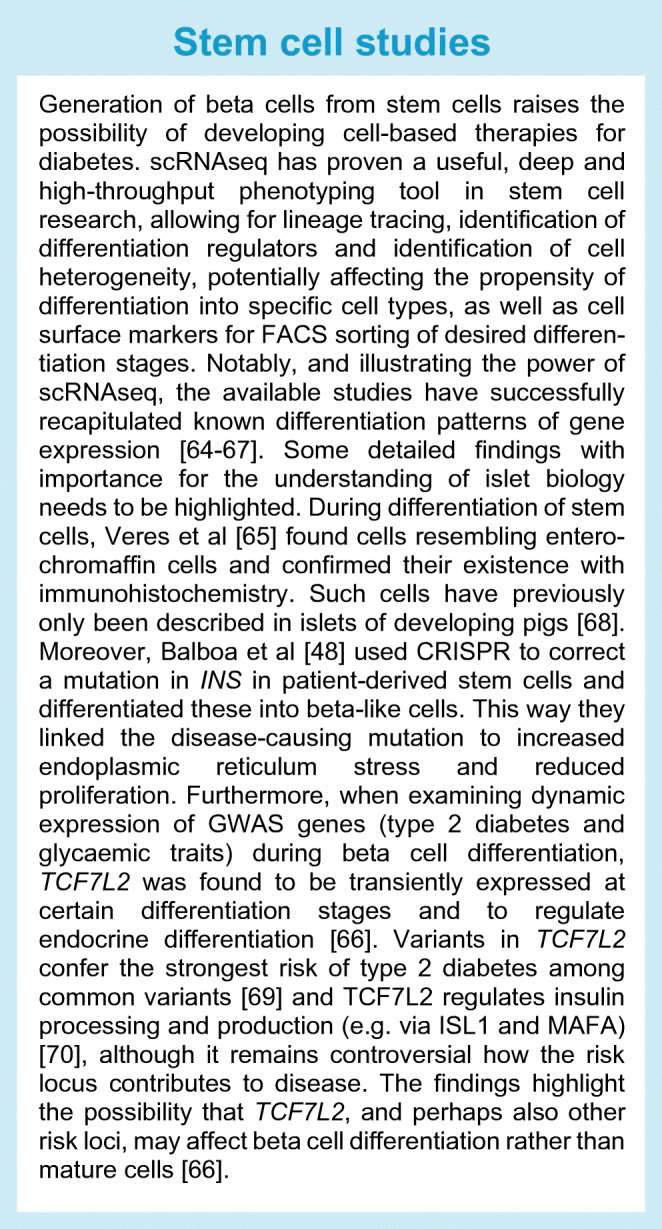







## Introduction

Impaired islet function is central in type 2 diabetes but the underlying mechanisms are not fully understood [1, 2]. The islets consist of beta, alpha, delta, PP and ghrelin cells (also known as epsilon cells), producing insulin, glucagon, somatostatin, pancreatic polypeptide and ghrelin, respectively [3]. Experiments in animal models have provided important contributions to our understanding of islet pathophysiology in type 2 diabetes. Access to human islets from organ donors has enabled experiments that have increased our understanding of human islet function substantially despite the inherent limitation of impact of isolation and culture procedures, as well as often limited information on medical history [4–6]. In the past decade single-cell RNA sequencing (scRNAseq) has impacted several disciplines within biology and facilitated cataloguing of cell types in entire tissues and organisms [7–9]. The ability to profile individual cell transcriptomes is powerful and in recent years the technology has gained unprecedented adoption over bulk RNA sequencing (bulkRNAseq). Given its higher efficiency in capturing RNA transcripts, scRNAseq has made transcriptome profiling of rare cell populations possible despite the inherent limitation in the amount of starting material. The method enables single-cell resolution of gene expression parameters (e.g. variability, shape of gene expression distribution and transcriptional kinetics) that would be masked in bulkRNAseq [10]. Most tissues and cell populations are heterogeneous and scRNAseq provides a robust approach for characterising this heterogeneity. The technique is also invaluable in defining how distinct populations of cells may respond to stimuli (e.g. treatment, surroundings, etc.). Another key application of scRNAseq is the prediction of cellular progression along dynamic processes, allowing for detection of key drivers of the process and possibly allowing tracing (e.g. path from progenitors to mature lineages).

Despite the remarkable progress in scRNAseq technology, significant improvements are still needed. Most scRNAseq protocols capture 5–20% of the transcripts in a cell, with the most sensitive full-transcript-length-coverage protocols capable of capturing up to 40% [11]. This requires several cells to be profiled to overcome technical variability and attain sufficient statistical power. It is typical to detect clusters in scRNAseq studies and the bioinformatic workflow often involves gene selection, dimensionality reduction and cluster discovery, culminating in a cell–cell distance measure. This distance tends to become ambiguous as the number of gene features increases due to the ‘curse of dimensionality’. Furthermore, analysis of dynamic processes often culminates in trajectories that are mere in silico predictions that must be complemented with biological knowledge for validation and directionality. Interestingly, the discovery that 15–25% of reads mapped towards intronic gene regions in most scRNAseq protocols enables inference of previous or expected states of an individual cell based on RNA velocity prediction [12, 13]. This helps to overcome the assumption of ergodicity that underpins most density-based trajectory methods.

Analysis of scRNAseq data is a rapidly evolving field when it comes to development of computational tools to utilise the full potential of the technique. The continued advancements in computational tools as well as scRNAseq protocols have the potential to significantly increase our understanding of disease mechanisms in complex tissues. In the last 5 years, several scRNAseq studies on human and mouse islets, as well as on stem-cell-derived islet-like cells, have been presented and here we will attempt to summarise how scRNAseq has increased our understanding of human islet biology and the challenges that remain. We also suggest potential ways forward that could further improve the utilisation of scRNAseq.

## Studies in islets from donors without diabetes

To date, 19 studies [14–32] have presented scRNAseq data on human islets, with varying characteristics, including sequencing depth, cell number and number of donors (electronic supplementary material [ESM] Table 1). Seven studies [14–19, 32] have compared samples from donors with and without type 2 diabetes and are discussed below. Overall, the studies show that scRNAseq is technically feasible in human islets and that there are pros and cons for the different protocols used. FACS-based cell capture methods (e.g. Smart-seq2) yield an under-representation of beta cells and an over-representation of alpha cells when compared with native islets [17]. This bias seems less apparent for the Fluidigm C1 method [14, 16, 18], which appears to be more prone to generating doublets (i.e. erroneous sequencing of two cells) [25]. To date, two studies [19, 31] have utilised Drop-seq, a notable feature being the low number of ghrelin cells [19]. Moreover, single-nucleus RNA sequencing was recently shown to be a reliable approach for transcriptomic profiling of frozen islets, thus presenting the opportunity for use of archived islet material [29]. Different methods for cell type clustering achieved comparable results and accurately clustered cells into the expected pancreatic cell populations. This is likely attributed to the high mRNA content of the main hormonal product of each cell type (50% of the total transcripts for beta, delta and PP cells) [17]. To date, there have been no big surprises with respect to cell types and no evidence for novel cell types has been reported.

In a comprehensive assessment of five studies, Mawla et al [33] showed that many beta cell marker genes are only detected in fractions of beta cells. Although scRNAseq is not zero-inflated [34], absence of expression of single genes needs to be interpreted with caution as the risk of a false-negative result is high [24]. Nevertheless, scRNAseq has generated road maps of cell-type-specific gene expression in an unprecedented manner, providing information (e.g. expression of receptors, transcription factors, cell surface markers for sorting by FACS, and genome-wide association study [GWAS] genes) that could guide future experimentation and shed light on the cellular basis for genetic risk. Perhaps one of the most significant contributions is the characterisation of the rarer islet cell types, delta, PP and ghrelin cells, which have not hitherto been possible to sort by FACS [17, 24].

After confirming that islets exhibit the expected cell-type composition in agreement with established models based on histological studies, much attention has focused on subpopulations of cells, particularly of beta cells. It has long been known that not all beta cells are equal [35–37], and with the advent of scRNAseq there were high hopes of gaining insights into transcriptomic differences between beta cell subpopulations. Six studies report subpopulations of beta cells [15, 17, 19–21, 27], while others do not [16, 18, 25]. Mawla and Huising [33] compared genes reported to drive the subpopulations in five studies and found surprisingly little consensus, with not one single gene being replicated between all studies. It is still not known whether scRNAseq-derived subpopulations are, as discussed in [33, 36], a consequence of low detection rate by the technique leading to an artefactual overestimation of heterogeneity among cells. It is worth mentioning that clustering into subpopulations is dependent on the choice of method and the cut-offs selected, as shown in three islet datasets [38]. Furthermore, the extent to which subpopulations are driven by donor differences remains to be determined. With one exception [17], the reported studies do not specify influence of donors. Thus, observations of heterogeneity based on scRNAseq data should be carefully validated, either histologically (preferably in native pancreatic tissue to rule out influence of the isolation procedure) or in live cell experiments allowing for correlation of gene expression with functional readouts. An elegant example of the latter is a study from MacDonald’s group [15], who sequenced cells after thorough electrophysiological characterisation (a technique known as patch-seq). Using this approach they linked cellular functional properties, including exocytosis, to differences in the transcriptome and found that beta and alpha cells exhibited significant transcriptomic and electrophysiological heterogeneity. Furthermore, by using 484 genes that were highly expressed and correlated with electrophysiological parameters, the functional properties of a beta cell could be predicted.

Several important findings made possible by scRNAseq need to be highlighted. Fang et al [19] found 1188 beta cell genes associated with obesity and Segerstolpe et al [17] reported genes (e.g. *PCSK1N*) with cell-type-specific correlation to BMI. Several of the cell-type-resolved, BMI-correlated genes did not correlate with BMI when using a simulated bulk analysis [17]. By studying islets from donors spanning from 1 month to 54 years of age, Enge et al [22] found that transcriptional noise increases with age and associates with cellular stress. In addition, somatic mutations increased with age (both in alpha and beta cells but particularly in duct cells) and, as a sign of potential cellular fate drift, atypical (double) hormone expression increased with age [22]. It should be mentioned that not many donors were used for each age and larger follow-up studies are warranted. Furthermore, Wang et al showed that alpha and beta cells from juvenile donors (19 and 24 months of age) differ from adult cells, indicative of incomplete differentiation [14]. As an example, a large fraction of genes enriched in adult beta cells were found to be expressed in juvenile alpha cells. These findings were corroborated, and a refined analysis of the postnatal islet maturation process was presented, in a significantly larger study from the same group [32].

Evidence for proliferating alpha cells has been demonstrated [14, 17–19, 26]. Dominguez Gutierrez et al [26] found that alpha cells proliferate at a fivefold higher rate than beta cells from the same donors and verified such cells with in situ hybridisation. Pseudotime analysis revealed a progressive increase in cell cycle score along the proliferating subpopulation state and several genes important for regulating alpha cell proliferation were put forward. The authors suggest replication of existing alpha cells as a source of renewal of alpha cells in adulthood [26]. On the same note, Li et al [30] and Marquina-Sanchez et al [31] used scRNAseq for assessing artemether-induced effects primarily on alpha cell gene expression.

Xin et al [27] identified beta cells clusters, with varying expression of *INS* and endoplasmic reticulum stress genes. Using pseudotime analysis, the authors proposed that beta cells transition between a state of activity with high *INS* expression and a state of recovery with low of *INS* expression and increased unfolded protein response activation [27]. In another study, Muraro et al [20] identified cell-type-enriched surface markers and tested their usefulness for sorting of alpha and beta cells using FACS. Improved alpha cell yield was seen when using CD24 and CD44 for sorting out acinar and ductal cell, respectively. *TM4SF4* was found to be highly enriched in alpha cells and an 85% pure alpha cell population was achieved when sorting for its encoded protein (transmembrane 4 L6 family member 4; TM4SF4) [20]. In another study, based on the same dataset, a protocol for scRNAseq data-guided sorting by FACS without antibodies was developed [39].

It is not well known how pancreatic cells are renewed and from which cellular source. Using a novel computational approach, StemID, Grün et al [28] identified subpopulations of pancreatic duct cells as potential multipotent cells. Notably, a subpopulation expressing *FTH1* and *FTL* was suggested to differentiate into beta cells and co-expression of ferritin light chain (FTL) and insulin in human ductal epithelial cells was confirmed immunohistochemically. The finding that StemID-identified *Lgr5*-expressing cells in the intestine of mice, as well as known stem cell populations in bone marrow, lends credibility to the pancreatic data. The concept of duct cells as progenitors gains support from a study on sorted duct cells [40]. Notably, when such cells were transplanted under the kidney capsule of immunodeficient mice, differentiation along all pancreatic lineages was observed.

Combining the sequencing depth and low cost of bulkRNAseq with the resolution of scRNAseq, using deconvolution for cell type adjustment is an attractive idea. Baron et al [21] developed a bulk sequence single-cell deconvolution analysis pipeline (Bseq-SC) and used their scRNAseq data for deconvolution of a published bulkRNAseq dataset [41]. Notably, many of the previously reported HbA_1c_-level-associated genes were not significantly associated after cell type adjustment. Further studies, preferably using scRNAseq and bulkRNAseq data from the same donors, are warranted to understand the potential and reproducibility of this approach.

Mouse models have been instrumental for our understanding of islet biology and evaluation of how well mouse islets reflect human islets was assessed in two studies [18, 21]. When comparing all genes, strong correlations were seen between non-diabetic human expression and expression of mouse homologous genes for both alpha and beta cells [18, 21]. However, for genes enriched in alpha or beta cells there were considerable differences between the two species [18]. For a summary of scRNAseq studies in mouse islets see Text box 1. These findings highlight the need for human islet experiments and the need for further evaluation of how well mouse models, including type 2 diabetes models, reflect the situation in humans.

## Studies in islets from donors with type 2 diabetes

Understanding cell-type-specific alterations in biological processes in type 2 diabetes will promote increased understanding of not only type 2 diabetes disease mechanisms but also potential compensatory mechanisms trying to counteract the disease. Seven studies have provided cell-type-specific information in donors with type 2 diabetes and non-diabetic donors [14–19, 32]. Studies performed in islets from donors with type 1 diabetes are summarised in Text box 2. Below, we provide a brief summary of the type 2 diabetes-related findings highlighted by the authors.

Segerstolpe et al [17] noted differential expression (DE) of 76 genes in beta cells, with *INS* and *FXYD2* being among the downregulated genes in type 2 diabetic beta cells while *GPD2* and *LEPROTL1* were among the upregulated genes. One hundred genes were differentially expressed in alpha cells and five genes (e.g. *ISL1*) were differentially expressed in PP cells. Gene set enrichment analysis showed that genes responsible for mitochondrial energy metabolism and protein synthesis were downregulated in most cell types, whereas apoptosis and cytokine signalling genes were upregulated in donors with type 2 diabetes. Xin et al [18] found DE of 48 genes in beta cells, 54 genes in alpha cells, 119 genes in delta cells and 33 genes in PP cells. Most of these genes have roles in non-islet cell growth, or no known function in islets, whereas 8.5% have a known function in islets. Wang et al [14] presented data from three adult non-diabetic donors and two adult donors with type 2 diabetes. No systematic DE analysis was performed but gene expression profiles of type 2 diabetes alpha and beta cells were found to be reminiscent of their juvenile counterparts, partly due to elevated expression of cell cycle genes. The authors suggest that type 2 diabetic beta cells may not be able to maintain fully differentiated status and that their data support previous studies on partial dedifferentiation in type 2 diabetic beta cells. In a larger follow-up study, Avrahami et al expanded on these findings and presented gene sets associated with type 2 diabetes in alpha and beta cells [32]. Lawlor et al [16] could not detect type-2-diabetes-driven clustering but found cells clustered by donor identity. DE was detected for 248 genes in beta cells, 138 genes in alpha cells and 24 genes in delta cells. Among the affected beta cell genes, the authors highlighted lower expression of *STX1A* and higher expression of *DLK1* in type 2 diabetic donors. *CD36* expression was increased, while *GDA* expression was lower in type 2 diabetic alpha cells.

Taking advantage of a high number of sequenced cells, Fang et al [19] used a novel approach to identify disease-associated alterations. Using the regressing principle components for the assembly of continuous trajectory (RePACT) approach, they analysed disease-associated single-cell heterogeneity. Although the analysis was based on a limited number of type 2 diabetic donors (*n* = 3), a large number (1368) of type 2 diabetes-associated genes was identified in beta cells (e.g. *IAPP* and *CPE*). Many genes were affected similarly by type 2 diabetes and obesity, although others (e.g. *INS*) were increased in obesity but reduced in type 2 diabetes. Furthermore, using a CRISPR screen for insulin regulatory genes in MIN6 cells, 17 of the obesity- or type-2-diabetes-affected beta cell genes were found to regulate intracellular insulin content. Whether or not the limited overlap between the two approaches is due to species differences remains to be established. Nonetheless, the cohesion-loading complex and the NuA4/Tip60 histone acetyltransferase complex were shown to be novel regulators of insulin. Camunas-Soler et al [15] also used the function-to-gene-expression approach to assess type 2 diabetes affected genes. The expression of genes that were first shown to be correlated with insulin exocytosis in non-diabetic donors was subsequently assessed in donors with type 2 diabetes. Interestingly, evidence for a compensatory exocytosis response in beta cells from donors with type 2 diabetes was presented. Furthermore, *ETV1* was enriched in beta cells from donors with type 2 diabetes, increased *ETV1* expression was associated with reduced exocytosis, and knockdown of *ETV1* rescued exocytosis specifically in these beta cells.

Except for observations of fewer beta cells in two studies [16, 17], type 2 diabetes has not yet been clearly associated with altered cell-type composition or altered subpopulations of cells. Quite remarkably, even though very few ghrelin cells have been profiled, all studies but that of Wang et al [14] (four cells in one donor with type 2 diabetes) find ghrelin cells exclusively in non-diabetic donors. This was also the case in our dataset (Smart-seq2 data from six non-diabetic donors and six with type 2 diabetes; Gene Expression Omnibus accession number: GSE153855) and 75% lower ghrelin cell density was confirmed immunohistochemically in islets from donors with type 2 diabetes [42].

**Fig. 1:**
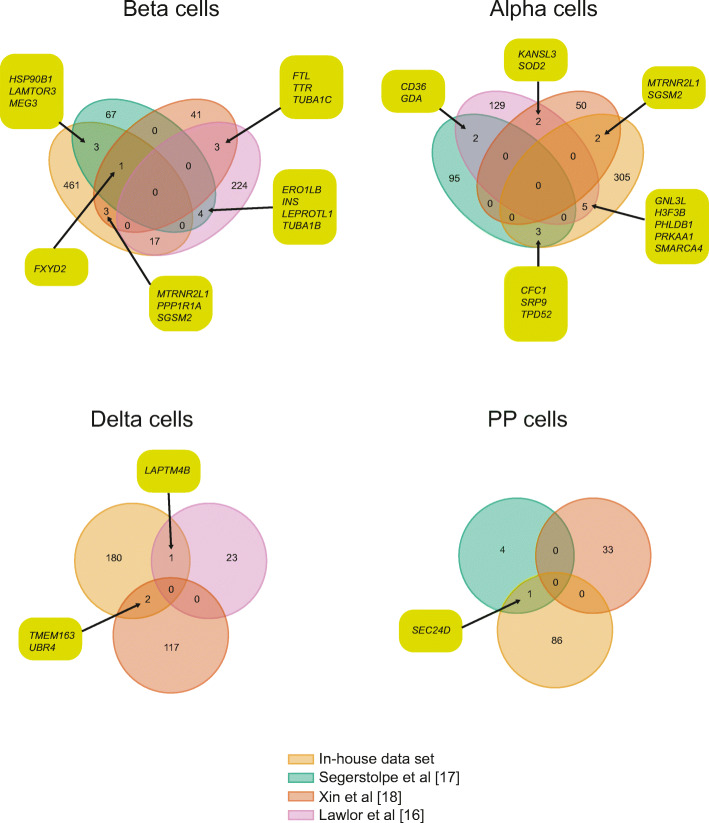
Differentially expressed genes in distinct islet cell types across four datasets, with genes overlapping in the datasets indicated. This figure is available as a downloadable slide

The ultimate goal of the studies comparing data from donors with and without type 2 diabetes was to identify type 2 diabetes disease mechanisms in beta cells. In three of the studies, systematic global DE analysis was performed in a comparable manner [16–18]. When comparing the differentially expressed beta cell genes for these studies with DeSeq2 [43] data from our dataset (GSE153855), the four studies had no differentially expressed genes in common, although *FXYD2* was differentially expressed in three studies and another 30 genes were shared between two studies (Fig. 1a). When including RePACT data from [19], *FXYD2* was the only gene common to four out of five studies and another 15 genes were common to three studies (ESM Fig. 1). A similarly low degree of overlap was seen for alpha, delta and PP cells (Fig. 1b–d).

A common disease mechanism or disease-induced alteration in gene expression would allow for replication across datasets. So far, DE of only one gene, *FXYD2*, has been convincingly replicated between studies. One interpretation of the data is that altered *FXYD2* expression is a major cause of beta cell dysfunction in type 2 diabetes. *Fxyd2* knockout mice exhibit improved glucose tolerance, beta cell hyperplasia and elevated fasting and postprandial plasma insulin levels [44]. However, *FXYD2* does not appear to be genetically associated with type 2 diabetes risk or beta cell function as one would expect if altered *FXYD2* expression was of major importance for beta cell dysfunction. An alternative interpretation is that the current approaches do not fully capture type 2 diabetes-induced disease biology. If they did, one would also expect genes with established and experimentally proven roles in beta cell pathophysiology to appear and for such genes to be replicated between studies. There are several reasons to suggest that this a likely interpretation: (1) the inherent low detection rate of scRNAseq could mean that disease biology is only captured in a fraction of the cells; (2) the available studies are small in terms of cell counts and participants, leading to low statistical power; (3) type 2 diabetes is a heterogeneous disease and five clear subtypes are defined [45], thus beta cells from an insulin-resistant individual, with cells capable of compensating for the insulin demand, will most likely behave differently from beta cells from an individual with a primary beta cell defect; (4) the data has been analysed using suboptimal methods for DE analysis [43]; and (5) the data lack adjustment for donor variance. In summary, the available studies find no association between type 2 diabetes and cell-type composition or subpopulations of islet cells. Individual reports put forward novel type 2 diabetes-associated genes, some of which have been functionally validated [15], but very few have been replicated between studies.

## Potential ways forward

The available studies have contributed important information that has increased our knowledge on many aspects of islet cell biology. However, with respect to identifying type 2 diabetes disease biology, the contribution to our understanding has been limited. We advocate that this is a consequence of DE analysis being a blunt tool for identifying alterations in biological processes between two groups in small-scale studies with heterogeneous starting material. scRNAseq data has proven to be highly heterogeneous, and the DE analysis tools attempt to overcome variability by averaging expression across cells. Likely this leads to a loss of meaningful information in lieu of very large sample sizes. A recent study highlighted the limitations of commonly used DE methods for scRNAseq data. Many of the methods were found to conflate variability between replicates with the effect of biological perturbations and a systematic bias towards highly expressed genes was noted [43]. Furthermore, DE analysis is based on the abundance of mRNA present in different cells. Recent advances suggest that the timing of mRNA expression, as well as spatial information on mRNA expression, provides meaningful information [46, 47]. Hence, the field needs refined computational tools to use the full potential of scRNAseq. We propose that an ultimate test of such tools is their ability to recapitulate known patterns of gene expression. There are examples of methods that hold promise for improved sensitivity and which have been able to recapitulate known gene expression patterns. Thus, trajectory-based methods (e.g. pseudotime analysis) have proven useful for studying gradual alterations towards different cellular states (e.g. cell lineages) and subpopulations of alpha and beta cells [26, 27, 37, 48]. This type of analysis involves first categorising cells into different states and thereafter ordering the cells onto trajectories based on their way through the process to another state. Caution is needed, as this method could span all cells with trajectories, regardless of whether they actually participate in a dynamic process [47]. RNA velocity is another approach that, based on the ratio of exon-to-intron reads, leverages the fact that newly transcribed, unspliced mRNAs can be distinguished and infers a time derivate of the gene expression state. Thereby, RNA velocity predicts the future state of individual cells on a time scale [13]. In the islet field, so far, RNA velocity (scVelo) has been used to show lineage relationships between islet cells in fetal mice and has outperformed pseudotime analysis in recapitulating known lineage relationships [12]. Both methods infer a time factor, based on the assumption that different cellular states represent different times on a time scale. In addition, both approaches exploit cellular heterogeneity, rather than averaging it as in DE analysis. On the same note, RePACT analysis has proven useful for identifying disease-associated cellular heterogeneity [19]. The method is based on organising cells into pseudostates along a type 2 diabetes trajectory (i.e. the degree to which a cell has transformed during disease development). Thereafter, cells from different pseudostates are compared. The advantage of this approach is that it does not assume that all cells are equally affected by disease and could potentially provide enhanced sensitivity for assessing disease-associated alterations; replication of its usefulness in larger studies and across datasets is warranted.

Notably, most approaches used to analyse scRNAseq data do not account for the possibility that gene expression could be organised and synchronised in genetic programmes (e.g. for orchestrating specific cellular functions). Network analysis has been successfully used to identify such programmes or modules (e.g. by assessing co-expression of mRNAs within a population of cells). Multiple techniques with varying capability of identifying disease-relevant pathways [49] or known regulatory networks [50] are available. Furthermore, methods for comparing changes in network structure between cells of two states (e.g. disease vs healthy) have also been developed. Interestingly, differential network analysis (DiNA), which enables detection of changes in the interplay between mRNAs rather than assessing changes in single mRNAs, has been shown to outperform DE analysis [51]. DiNA has, to the best of our knowledge, not been applied to scRNAseq data but combining this approach with a reliable method for dealing with donor variation seems attractive and should be evaluated and replicated in independent datasets.

In addition, increased numbers of samples and sequenced cells are needed to increase the analytical power and, likely, numbers approaching the ones presented in a recent bulkRNAseq study [52] are needed to increase the diversity of donors enough to allow for studies of different subtypes of diabetes [45]. One way of achieving this is to integrate existing datasets, as recently shown [53]. However, given the risk for influence from batch effects, large-scale studies with uniform sampling and analysis protocols with freshly isolated islets from well-characterised donors (e.g. clinical data, medical records, genome sequencing) is warranted.

## Concluding remarks

scRNAseq has generated unprecedented insight into important aspects of islet biology, foremost by uncovering cell-type-specific gene expression in all islet cell populations. Using the derivate of such data, novel computational methods have convincingly reproduced known biological processes (e.g. cell lineage tracing) in developmental and stem cell studies. When it comes to increased understanding of type 2 diabetes disease mechanism, it is likely we have still not seen the full potential of the technique. To move forward there is a need for the following: (1) large-scale studies with high-quality islets from well-characterised donors; (2) improved analysis methodology, capable of adjusting for donor variance and for exploiting the high dimensionality of scRNAseq data to untangle the biological processes that are altered in type 2 diabetes; (3) replication in independent datasets; and (4) experimental and histological validation of findings. Clearly, scRNAseq technology is here to stay and with the rapid development in the field, with respect to analysis tools and protocols with lower costs, the technique will most likely, within the near future, contribute to a major leap forward in our understanding of the altered characteristics of each islet cell type in type 2 diabetes.

## Supplementary information

ESM 1 (PDF 477 kb)

Figure slide (PPTX 170 kb)

ESM 3 (XLSX 16 kb)

## Abbreviations

bulkRNAseq: Bulk RNA sequencing
DE: Differential expression
DiNA: Differential network analysis
GWAS: Genome-wide association study
PP cells: Pancreatic polypeptide cells
RePACT: Regressing principle components for the assembly of continuous trajectory
scRNAseq: Single-cell RNA sequencing
