# Supplementary material for: Lessons from single-cell RNA sequencing of human islets
Source: Diabetologia. 2022 Apr 28;65(8):1241–50. doi: 10.1007/s00125-022-05699-1 (PMC9283180; doi:10.1007/s00125-022-05699-1)

ESM figure1. Differentially expressed genes in distinct islet cell types across datasets.

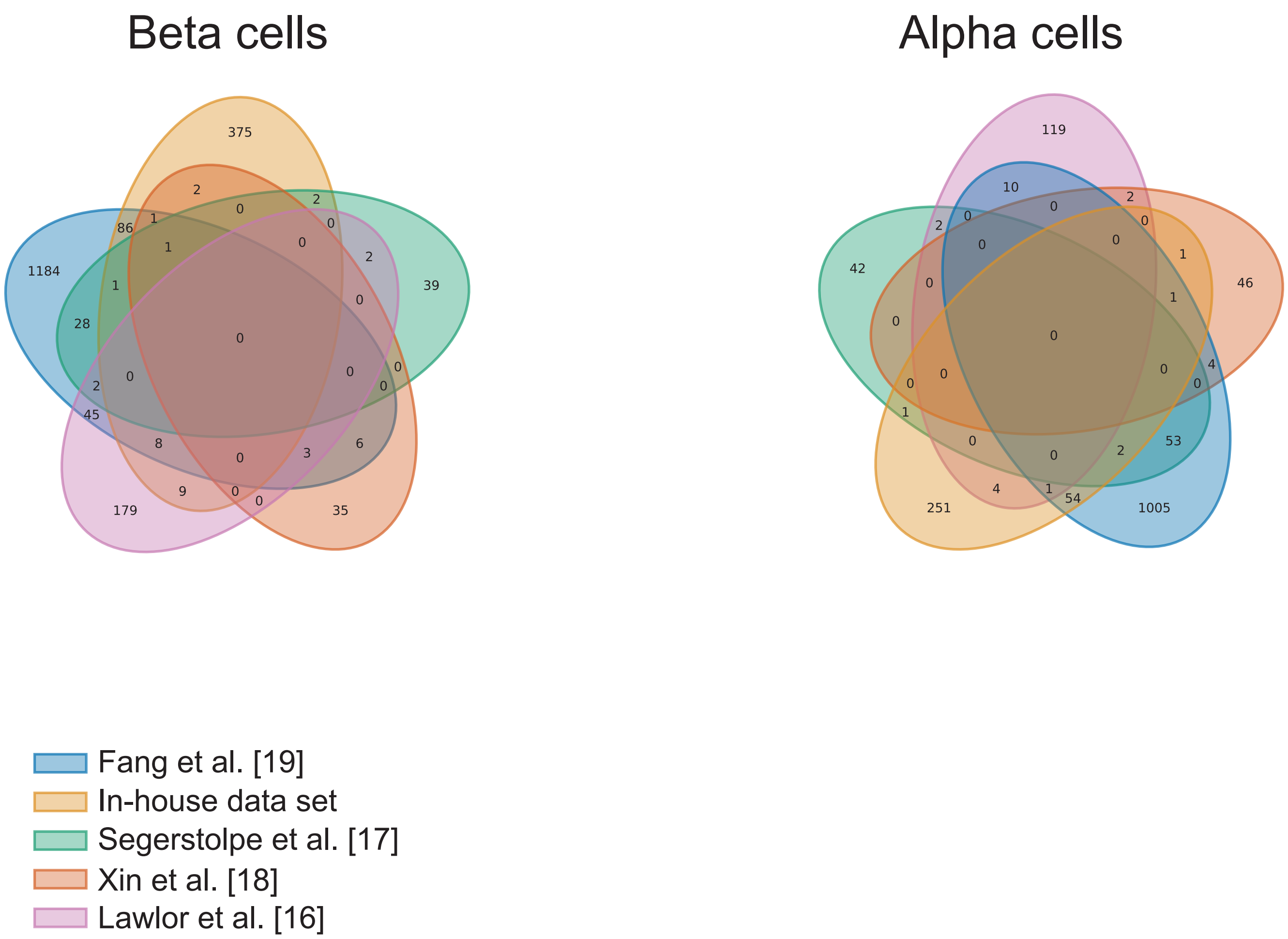

Supplement: Supplementary file 1 — (PDF 477 kb) [file 125_2022_5699_MOESM1_ESM.pdf]
