# Supplementary material for: Lessons from single-cell RNA sequencing of human islets
Source: Diabetologia. 2022 Apr 28;65(8):1241–50. doi: 10.1007/s00125-022-05699-1 (PMC9283180; doi:10.1007/s00125-022-05699-1)
Supplement: Supplementary file 2 — (PPTX 170 kb) [file 125_2022_5699_MOESM2_ESM.pptx]

## Slide 1
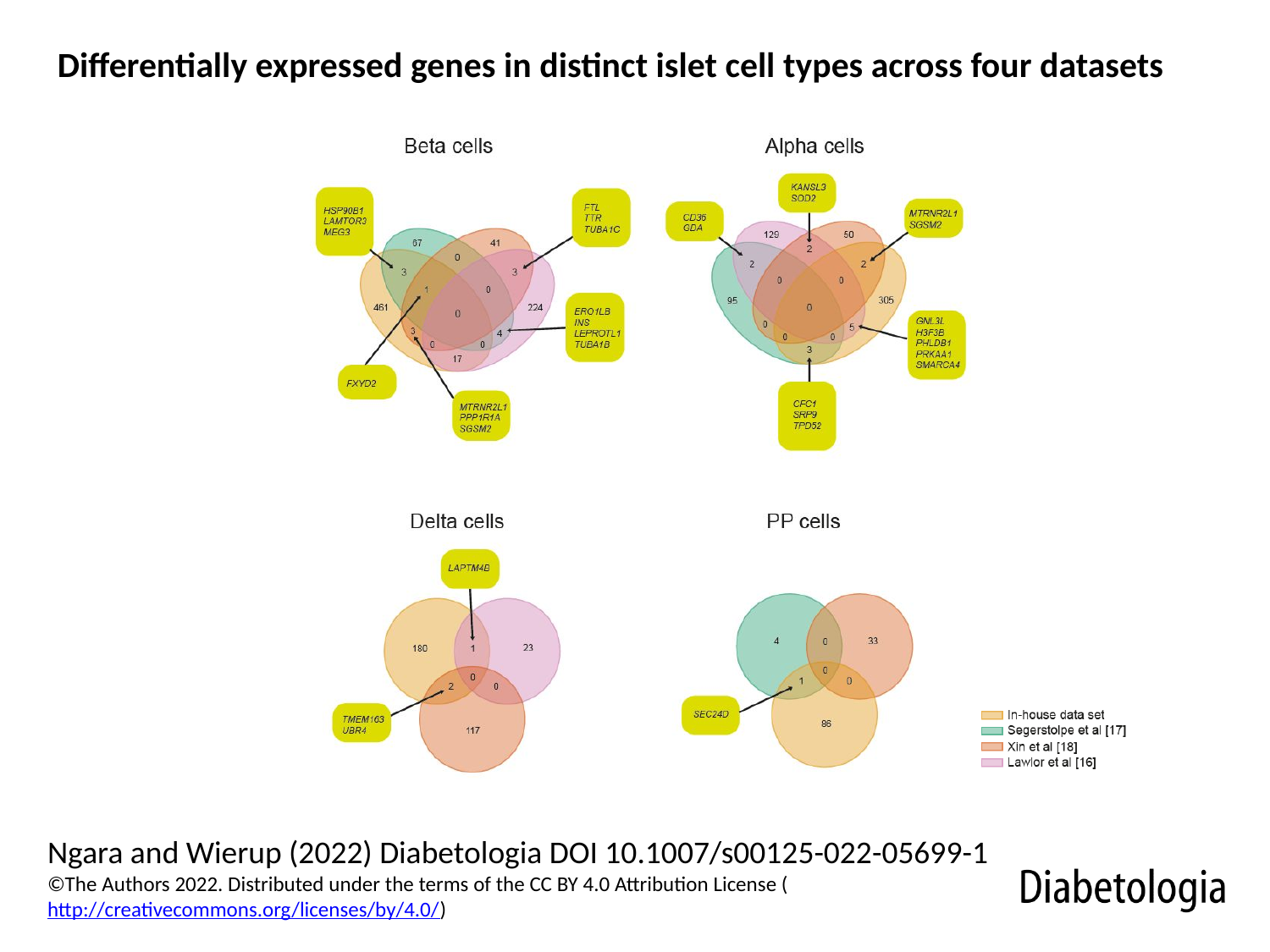

Differentially expressed genes in distinct islet cell types across four datasets
Ngara and Wierup (2022) Diabetologia DOI 10.1007/s00125-022-05699-1
©The Authors 2022. Distributed under the terms of the CC BY 4.0 Attribution License (http://creativecommons.org/licenses/by/4.0/)
